# The complete mitogenome of *Pentacheles validus* (Decapoda: Polychelidae)

**DOI:** 10.1080/23802359.2018.1455160

**Published:** 2018-05-25

**Authors:** Xiaofeng Shi, Dingyong Huang, Jianjia Wang, Wentao Niu, Rongcheng Lin

**Affiliations:** Laboratory of Marine Biology and Ecology, Third Institute of Oceanography, State Oceanic Administration, Xiamen, PR China

**Keywords:** *Pentacheles validus*, mitogenome, evolutionary, next-generation sequencing

## Abstract

In this study, the complete mitogenome sequence of *Pentacheles validus* has been decoded for the first time. The overall base composition is 33.4% for A, 25.0% for C, 10.8% for G, and 30.9% for T and has low GC content of 35.8%. The assembled mitogenome, 16,079 bp in length, has unique 13 protein-coding genes (PCGs), 22 transfer RNAs and 2 ribosomal RNAs. The mitogenome shares 80% identity to *Polycheles typhlops*. The complete mitogenome of *P. validus* provides essential DNA molecular data for further phylogenetic and evolutionary analysis for Polychelidae and deep-sea faunas.

The deep-sea lobster family, Polychelidae are one of the most characteristic families in deep-sea faunas with strongly reduced eyes (Torres et al. 2014). However, species of the family have been in a constant state of taxonomic confusion, especially in the case of *Pentacheles* (Joel et al. 2009). Mitochondrial genome sequences have become the most important tool in molecular taxonomy and hypotheses on evolution (Lynch et al. 2006). Nevertheless, no complete mitochondrial genome sequences of *Pentacheles*, to date, have yet been available. In this study, we determined the complete mitochondrial genome sequence of *Pentacheles validus*, which is generally collected from unknown habitat at a depth ranging from 914 to 3365 m. To date, little is known regarding this species.

Samples of *P. validus* were collected from the Southwest Indian Ridge (SWIR) (geographic coordinate: S 37°51’ 36”, E 49°46’ 51.6”). The methods for genomic DNA extraction, library construction, and next-generation sequencing were described in previous publication (Shen et al. 2016). The whole body specimen was preserved in ethanol and deposited in Laboratory of Marine Biology and Ecology of Third Institute of Oceanography, State Oceanic Administration. Samples are available on request. The complete mitogenome of *P. validus* contains 16,079 bp in size (GenBank MH011414) with overall base composition of 33.4% for A, 25.0% for C, 10.8% for G, and 30.9% for T. It has low GC content of 35.8%, showing 80% identities to the complete mitogenome of *Polycheles typhlops* (GenBank KC107818) (Shen et al. 2013).

The protein coding genes, rRNA and tRNA of *P. validus* mitogenome were predicted with DOGMA (Wyman et al. 2004), ARWEN (Laslett and Canback 2008), and MITOS (Bernt et al. 2013) tools, and manually inspected. The complete mitogenome of *P. validus* includes unique 13 protein-coding genes (PCGs), 22 transfer *RNA* genes, and two ribosomal *RNA* genes together with a putative control region. In the complete mitogenome, 4 PCGs (*ND1*, *ND5*, *ND4,* and *ND4L*), 2 rRNA (12S rRNA and 16S rRNA), and 8 tRNA (*tRNA-Val*, *tRNA-Leu*, *tRNA-Tyr*, *tRNA-Phe*, *tRNA-Gln*, *tRNA-Cys*, *tRNA-Pro*, and *tRNA-His*) genes are encoded on L-strand, while other genes are encoded on the H-strand. The *P. validus* mitogenome has similar gene organization and feature to *Polycheles typhlops*. It is important to note that *COX2* and *ND5* had incomplete stop codons which would be presumably completed as entire stop codon (TAA) via post-transcriptional polyadenylation.

To validate the phylogenetic position of *P. validus,* we used MEGA7 software (Pennsylvania State University, State College, PA) (Kumar et al. 2016) to construct a maximum likelihood (ML) tree (with 1000 bootstrap replicates) containing complete mitogenomes of 11 species derived from Decapoda. The sea slater, *Ligia oceanica*, derived from Isopoda was used as outgroup for tree rooting. According to the tree topology, *P. validus* is close to *Polycheles typhlops*. These two species formed a monophyletic group (Figure 1). In conclusion, the complete mitogenome of the *Pentacheles validus* deduced in this study provides essential and important DNA molecular data for further phylogenetic and evolutionary analysis for Decapoda phylogeny.

**Figure 1.**
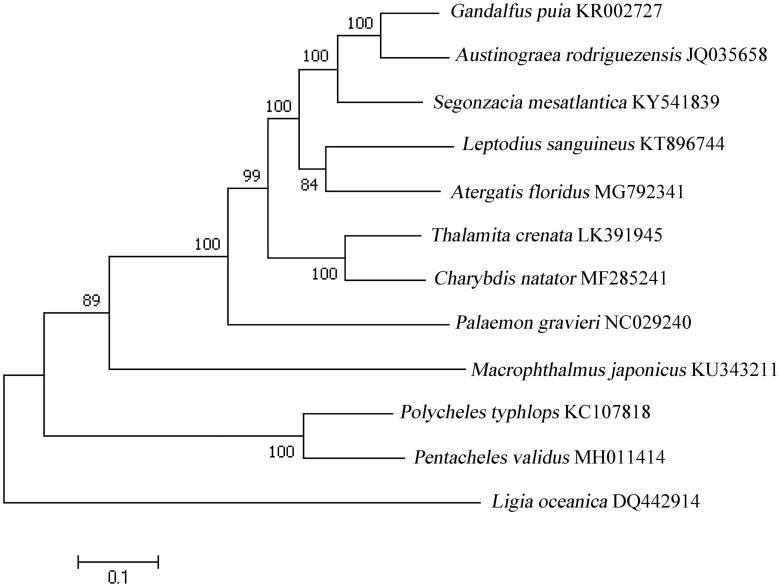
Molecular phylogeny of *Pentacheles validus* mitochondrial genome based on the maximum likelihood (ML) method using a Kimura 2-parameter model. The bootstrap values are shown at node branches (>50). Initial tree(s) for the heuristic search were obtained automatically by applying Neighbour-Join and BioNJ algorithms to a matrix of pairwise distances estimated using the maximum composite likelihood (MCL) approach, and then selecting the topology with superior log-likelihood value. The tree is drawn to scale, with branch lengths measured in the number of substitutions per site. The analysis involved 12 mitochondrial genome sequences.
